# Pleasant freedom with suffering wounds: a qualitative study of post-discharge life in trauma patients recovered from coma

**Published:** 2026-01

**Authors:** Asad Imani, Shahram Molavynejad, Mojgan Khademi, Mohammad Adineh, Mohsen Savaie

**Affiliations:** ^*a*^ Student Research Committee, School of Nursing and Midwifery, Ahvaz Jundishapur University of Medical Sciences, Ahvaz, Iran.; ^*b*^ Nursing Care Research Center in Chronic Diseases, School of Nursing and Midwifery, Ahvaz Jundishapur University of Medical Sciences, Ahvaz, Iran.; ^*c*^ Community-Oriented Nursing Midwifery Research Center, Shahrekord University of Medical Sciences, Shahrekord, Iran.; ^*d*^ Social Determinants of Health Research Center, School of Nursing and Midwifery, Lorestan University of Medical Sciences, Khorramabad, Iran.; ^*e*^ Pain Research Center, Ahvaz Jundishapur University of Medical Sciences, Ahvaz, Iran.

**Keywords:** Coma, Unconsciousness, Trauma, Injuries, Intensive Care Units, Discharge

## Abstract

**Background:**

Trauma patients recovering from coma face significant physical, psychological, and social challenges after returning home, which may adversely affect their daily lives and well-being. Exploring these experiences can inform care needs and guide tailored post-discharge support. Accordingly, this study aimed to explore post-discharge life experiences in trauma patients recovered from coma.

**Methods:**

This qualitative study was conducted using conventional content analysis. A total of 17 trauma patients who had recovered from coma and had been discharged at least three months prior were selected through purposive sampling. For data collection, 20 face-to-face, semi-structured interviews were conducted until data saturation was achieved. Data analysis followed the Graneheim and Lundman approach. The rigor of the study was ensured using Guba and Lincoln’s criteria.

**Results:**

The findings revealed that the main theme of post-discharge life experience was characterized as “pleasant freedom with suffering wounds,” encompassing three categories: “freedom from captivity of alienation and despair,” “dubious pleasure,” and “integration of real and imagined disability and helplessness.”

**Conclusions:**

The initial perception of post-discharge life among trauma patients who recovered from coma is profoundly shaped by the unpleasant experiences endured during ICU hospitalization. The sufferings experienced at home reflect a combination of real complications and imagined concerns arising from the severity of trauma, often accompanied by a false sense of disability and helplessness. These findings underscore the necessity of softening the ICU environment, implementing comprehensive discharge preparation, and providing effective post-discharge follow-up that addresses not only actual physical and psychological problems but also perceived or imagined sufferings.

## Introduction

Trauma is one of the leading causes of death worldwide^1^ and constitutes a major public health concern due to its extensive physical, psychological, and social consequences for patients and their families.^2^ A subset of trauma patients experience coma during the acute phase of injury, and the coexistence of severe trauma and coma substantially complicates the recovery process. Beyond physical impairments, these patients often face significant psychological challenges, including loneliness,^3^ post-traumatic stress and anxiety,^4^ and social withdrawal after regaining consciousness.^5^

In recent years, advances in trauma management and intensive care medicine have increased survival among critically ill trauma patients, including those who experience prolonged coma.^6^ Consequently, clinical and research attention has shifted from survival alone toward understanding recovery trajectories and quality of life after hospital discharge. Post-discharge life represents a particularly vulnerable period during which patients and their families must adapt to new care demands, uncertainty regarding prognosis, and altered personal and social roles.^7^

During this period, caregivers’ perceived inability to provide adequate care and the anticipated difficulty or even impossibility of caregiving can generate substantial stress, especially in the early days following discharge.^8^ Understanding patients’ lived experiences after discharge is essential for anticipating unmet needs, preventing avoidable complications, and promoting recovery.^9^ Previous studies have shown that post-discharge experiences can influence patients’ physical and psychological well-being^10^ as well as the quality of life of both patients and their caregivers.^11^ Despite this, most existing literature has predominantly focused on in-ICU experiences,^12^ psychological distress of family members^13^ or the professional experiences of ICU nurses.^14^ Empirical evidence specifically addressing the post-discharge experiences of trauma patients who have recovered from coma remains limited. In the present study, “post-discharge life” is operationally defined as the experience of returning to one’s own home following discharge from the hospital after ICU and ward hospitalization due to trauma-related coma.

By exclusively exploring the post-discharge experiences of trauma patients who have recovered from coma as a distinct population, this study addresses an important gap in the literature. The findings provide timely evidence to inform patient-centered discharge planning, follow-up care, and supportive interventions. Accordingly, the primary aim of this qualitative study was to explore the post-discharge life experiences of trauma patients who had recovered from coma.

## Methods 

**Study Design and Research Arena**

This study employed a qualitative research design using conventional content analysis to explore the post-discharge experiences of trauma patients who had recovered from coma. This approach was selected due to the exploratory nature of the study and the limited prior knowledge regarding the phenomenon. The study was conducted at Golestan Teaching Hospital, affiliated with Ahvaz Jundishapur University of Medical Sciences, in southwest Iran, from 2022 to 2025. This hospital operates within a trauma care system in which structured post-discharge follow-up and transitional care services are limited. Consequently, after ICU discharge, most patients rely primarily on family members for home care, with minimal professional support.

**Participant Eligibility Criteria**

Out of 30 eligible patients, 25 were approached, 8 declined to participate, and 17 were finally enrolled in the study according to the following inclusion criteria:

- Hospitalization in the trauma ICU for at least 10 days.

- Glasgow Coma Scale (GCS) ≤ 8 for a minimum of 5 consecutive days during ICU stay. (True coma duration was extracted from patients’ discharge records, and days during which depressed consciousness was attributable to full sedation with medications were excluded)

- Discharged to home from the ICU at least 3 months prior to recruitment.

- Sufficient alertness and cognitive function to participate in interviews. (To ensure participants’ ability to provide reliable narratives, there were clinically assessed by the researchers prior to the interview using simple orientation questions regarding time, place, and person)

- Absence of diagnosed cognitive disorders.

- Willingness to participate in the study.

**Data Collection**

Data were collected through individual, face-to-face, semi-structured interviews conducted by the first author (Asad Imani). The interviewer was a PhD nursing student with formal training in qualitative research methods and interviewing techniques. He had no prior therapeutic or professional relationship with the participants.

Each interview began with an open-ended question: “Please tell us about your experience of life after being discharged from the intensive care unit.” During the interviews, probing questions such as “Could you explain further?”, “What do you mean by that?”, “Can you give an example?”, and “Is there anything else you would like to add?” were used to elicit richer descriptions. Efforts were made to build rapport and gain participants’ trust by adhering to principles of active listening, empathy, non-judgmental interaction, and strict confidentiality.

A total of 20 interviews were conducted in private rooms of hospital or participants’ homes, depending on preference, and lasted approximately 45 minutes. Interviews were continued until data saturation was reached; no new codes or subcategories emerged after the 17th interview. To enhance the richness and depth of the data, repeated interviews were carried out with participants 1, 16, and 17.

**Ethical Considerations**

This study was endorsed on 31 August 2022 by Internal Review Board of Ahvaz Jundishapur University of Medical Sciences with allocated ethical code: (IR.AJUMS.REC.1403.284).

Prior to participation, the study objectives and significance were explained to all eligible participants, after which written informed consent was obtained for participation and audio recording of the interviews. All audio files were permanently deleted after verbatim transcription. The principle of anonymity was strictly observed at all stages of the study, particularly in reporting the findings.

**Data Analysis**

Data analysis and interpretation were conducted following the approach proposed by Graneheim and Lundman.^15^

Each interview was listened to several times and transcribed verbatim in MAXQDA 2020. Meaningful segments of participants’ statements were identified as condensed meaning units and labeled as initial codes by the first author. Following consultation with the research team, conceptually similar codes were grouped into subcategories, and related subcategories were further merged to form categories. These categories were then synthesized into main themes, representing a higher level of conceptual abstraction. Throughout all stages of the analysis, discrepancies were resolved through discussion and consensus within the research team.

 “In the early days after discharge, my face had changed; I had no hair, and my throat was open because of the tracheostomy. In that condition, I didn’t want anyone to see me, so I told my mother that no one except her and my husband was allowed into my room.” (P.7, a 32-year-old female)

 After discussion with the research team, it was determined that this code, along with other codes—such as wearing a hat to hide a bald spot (Participant 4), covering the thoracostomy site with a cloth (Participant 9), unwillingness to speak following a change in voice (Participant 10), being bothered by a deepening voice (Participant 11), unwillingness to be in public following facial changes (Participant 12), and not participating in family gatherings following facial changes (Participant 13)—fell under the subcategory “loss of ability to speak and facial changes as barriers to social interaction.”

This subcategory, together with three other subcategories— “Imagining Brain Trauma as a Severe and Permanent Disability,” “pain and hopelessness,” and “Feeling guilty about not fulfilling roles”—formed the category “Integration of real and imagined disability and helplessness.” This category reflects the suffering wounds characteristic of the main theme “Pleasant freedom with suffering wounds.”

**Rigor**

To ensure the rigor or trustworthiness of the study, the criteria proposed by Guba and Lincoln—credibility, dependability, confirmability, and transferability—were explicitly operationalized.^16^ Credibility was enhanced through maximum variation purposive sampling based on age, gender, length of ICU stay, duration of coma, and time since discharge. Prolonged engagement with the data was achieved through iterative data collection and analysis. Data saturation guided sampling decisions and was considered achieved when no new codes or subcategories emerged after the 17th interview. Member checking was conducted by returning preliminary codes and interpretations to selected participants to verify accuracy and resonance with their experiences.

Dependability was ensured through peer debriefing and coding checks. Initial coding was performed by the first author and subsequently reviewed by the research team. Coding decisions, category development, and analytical revisions were discussed in regular team meetings, and disagreements were resolved through consensus.

Confirmability was supported by maintaining a detailed audit trail, including raw data, coding frameworks, analytic memos, and documentation of decision-making throughout the analysis process. In addition, the research team engaged in reflexive discussions to critically examine assumptions and minimize researcher bias during data interpretation.

Transferability was facilitated by providing a rich description of the research context, participant characteristics, and analytic procedures, allowing readers to assess the applicability of the findings to similar settings and populations.

## Results

A total of 17 participants, including 10 males and 7 females, with diverse marital status, educational, and occupational backgrounds, were included in the study. The mean age of participants was 41.35 years (range: 22–63 years). The mean duration of ICU hospitalization was 28.82 days (range: 16–45 days), the mean duration of coma was 19.76 days (range: 9–33 days), and the mean time since discharge was 13.96 months (range: 6–22 months). Most participants had experienced trauma and coma as a result of car accidents (Table 1).

**Table 1:** Participants’ Characteristics.

Number	Gender	Age (Year)	Academic Degree	Job	Marital Statue	Number of Children	Trauma Type	Hospitalization Time (Day)	Coma Duration (GCS≤8) (Day)	Discharge Time (Month)
1	Male	22	Diploma	Self- employed	Single	-	Car Accident	16	9	6
2	Male	47	Bachelor	Labourer	Married	2	Falling Down	28	18	11
3	Female	35	Bachelor	Housekeeper	Married	1	Car Accident	30	14	14
4	Male	42	Bachelor	Teacher	Married	2	Car Accident	45	33	18
5	Male	49	Bachelor	Military	Married	3	Car Accident	32	20	9
6	Male	53	Bachelor	Teacher	Married	4	Car Accident	29	17	18
7	Female	32	Bachelor	Housekeeper	Married	1	Car Accident	25	16	13
8	Male	28	Bachelor	Athlete	Single	-	Car Accident	20	14	8
9	Female	45	Bachelor	Housekeeper	Married	2	Car Accident	22	15	16
10	Male	60	Elementary	Self- employed	Married	4	Car Accident	40	32	18
11	Female	52	Associate	Teacher	Married	3	Car Accident	32	20	15
12	Male	27	Bachelor	Self- employed	Married	-	Motor Accident	25	17	10
13	Male	25	Bachelor	Accountant	Single	-	Car Accident	40	28	22
14	Female	34	Bachelor	Hairdresser	Married	-	Car Accident	35	27	21
15	Female	63	Elementary	Housekeeper	Married	3	Car Accident	30	21	13
16	Male	58	Bachelor	Retired	Married	4	Car accident	31	18	14
17	Female	31	Bachelor	Self- employed	Single	-	Car Accident	27	17	11

Data analysis revealed that the emerging theme of post-discharge life experience was “pleasant freedom with suffering wounds,” which was reflected in three categories: “freedom from the captivity of alienation and despair,” “dubious pleasure,” and “integration of real and imagined disability and helplessness.” Further details are presented in Table 2. 

**Table 2:** Theme, Categories, Subcategories

Theme	Category	Subcategory
Pleasant freedom with suffering wounds	Freedom from captivity of alienation and despair	Feeling of captivity in the prison
		Feeling confused in an alien land
		Exit from darkness of despair
	Dubious pleasure	Pleasurable luck
		Restlessness and doubt about returning
	Integration of real and imagined disability and helplessness	Imaging brain trauma as a severe and permanent disability
		Pain and helplessness
		Feeling guilty about not fulfilling roles
		Loss of speech and altered appearance as barriers to social interaction

**1. Pleasant freedom with suffering wounds**

The main theme emerging from participants’ post-discharge experiences was “pleasant freedom with suffering wounds.” The findings revealed that returning home did not mark the end of pain and suffering; rather, patients were discharged while carrying persistent concerns that were either real or exaggerated by themselves and others. Although discharge was perceived as a highly pleasant and meaningful event, participants’ experiences showed that this sense of pleasure was deeply influenced with their distressing memories of the ICU and anxiety about returning to hospital conditions. Instead of focusing solely on life after discharge, participants continuously related their present experiences to the hardships endured during hospitalization. In this context, feelings of pleasure and freedom gained significance precisely because they were contrasted with the confinement, suffering, and unpleasantness experienced in the ICU. Accordingly, the main theme of “pleasant freedom with suffering wounds” was articulated through three interrelated categories:

**2. Freedom from captivity of alienation and despair**

Participants described post-discharge life as freedom from the captivity of alienation and despair, reflecting relief from the despairing and ambiguous burdens experienced during hospitalization. This category comprised three subcategories:

**2.1. Feeling of captivity in the Prison**

Participants described their ICU stay as an experience of involuntary confinement, frequently comparing it to being in prison. This feeling of captivity stemmed not only from physical and psychological distress but was also reinforced by care practices, particularly the use of physical restraints and limited attention to patients’ subjective expectations. Such conditions intensified patients’ sense of powerlessness and loss of autonomy. 

- " I hated being in the ICU so much that I felt like someone in prison counting the days until freedom. I had exactly the same feeling” (P6, a 53-year-old man).

*-“I was extremely restless because of a severe headache, but instead of paying attention to my pain, the nurse forcibly lowered my hand, gave me medication, and tied my hand to the bed. At that moment, I felt imprisoned” (P9, 45-year-old woman).*

Consequently, transfer to a general ward or discharge from the ICU was perceived as liberation, evoking strong feelings of freedom and relief.  Participants 1 and 2 described their experiences in this regard as follows:

*- "But the day I wanted to go to the general ward, it was as I was freed from the cage" (P. 2, a 47-year-old male).*

*- “After being discharged from the ICU, it was as if they had given me the whole world; it felt like I had been in prison and was finally set free” (P1, a 22-year-old man).*

**2.2. Feeling confused in an alien land**

Some participants experienced the trauma ICU environment as ambiguous, unfamiliar, and alien. A lack of orientation and insufficient communication about their condition, prognosis, and the ICU environment contributed to confusion, disconnection, and a sense of abandonment. The absence of explanatory interactions by healthcare providers intensified feelings of isolation and loss of meaning, leading participants to perceive the ICU as a foreign and unsettling place rather than a therapeutic environment.

*- “When I was in the ICU, I was confused. I didn’t know who I was or where I belonged. I didn’t know when I would be released from the ICU. I cannot describe it; it was a vague and unpleasant feeling” (P1, a 22-year-old man).*

Another participant described a profound sense of isolation:

*- “I was an abandoned person lost in an alien land, with no connection to the people around me” (P.5, 49-year-old male).*

**2.3. Exit from darkness of despair**

Participants’ experiences indicated that being in the ICU was often accompanied by profound feelings of despair. The intensity of this despair led some participants to describe the ICU environment as completely dark, both physically and emotionally. One participant expressed:

*- “Everything was dark in the ICU. You didn't know when you would get out of that situation. I had no hope of getting out of that darkness. When I left the ICU, it was like I escaped from darkness” (P.14, 34-year-old woman).*

**3. Dubious pleasure**

The unpleasant experience of confinement in the dark and alienating environment of the ICU eventually came to an end as patients were discharged to home. Although returning home was described as a pleasant and fortunate event, participants’ narratives revealed that this experience was accompanied by lingering doubt and worry, casting a shadow over the sense of relief. The following features further describe this complex emotional experience.

**3.1. Pleasurable Luck**

Participants described the moment of returning home as a profound source of happiness, viewing it as both a second chance at life and an escape from a critical and threatening situation. As one participant expressed:

*- “I can't express how happy I was after being discharged from the ICU. I can say that I still feel happiness from that day until now, even though more than a year has passed” (P.11, 52-year-old woman).*

Participants described returning home as a stroke of luck and a renewed chance at life. For them, survival after discharge from the hospital was not taken for granted; rather, it was perceived as something that could not have happened without fortune. One participant stated:

*- “In the discharge circumstance, if I hadn't been lucky, there's no doubt that I wouldn't have been able to return to normal life” (P.3, 35-year-old woman).*

**3.2. Restlessness and doubt about returning**

Despite the deep sense of happiness associated with surviving the ICU, participants’ discharge experiences were accompanied by persistent doubts and concerns. This sense of “Restlessness and doubt” reflects the emotional contrast between the fear and hardship of the ICU and the relief and gratitude associated with survival and homecoming.

 These worries stemmed from multiple sources, particularly the ongoing complications of trauma and coma, which made patients fearful of possible deterioration and readmission to the ICU. As one participant explained:

*- “I was terrified of going back to the ICU because of the complications after discharge” (P.13, 25-year-old man).*

This lingering restlessness highlights that the transition home, though pleasant, was overshadowed by uncertainty about the stability of their health and the possibility of facing the same critical conditions again.

**4. Integration of real and imagined disability and helplessness**

Participants’ experiences indicated that after hospital discharge, they faced significant and multifaceted concerns that diminished the pleasure of leaving the ICU and hospital. While many of these difficulties and limitations were real, some were exaggerated, stemming from patients’ own perceptions, imagination, or influence from others. As a result, concerns were often experienced as more severe than they actually were, and fear of persistent problems left patients feeling immobilized and powerless. Several key dimensions of this experience are described below:

**4.1. Imaging brain trauma as a severe and permanent disability**

Some participants held exaggerated perceptions of brain function and overestimated the long-term consequences of their injury. This unrealistic appraisal led them to believe they were severely disabled in terms of physical, cognitive, and social functioning. One participant reflected:

*- “After brain trauma, I thought my mind wasn't working properly anymore, I couldn't make the right decisions” (P.2, 47-year-old man).*

These perceptions were not solely the result of the patients’ own thoughts but were also reinforced by comments from relatives and acquaintances. As another participant recalled:

*- “I remember one day a relative who came to visit me said that he wished everything in my body was damaged except my brain, because the brain is the most versatile part of the body and it gives orders to do anything in any part of the body” (P.15, 63-year-old woman).*

Such messages intensified the belief that brain trauma inevitably leads to irreversible and global disability, reinforcing feelings of helplessness.

**4.2. Pain and helplessness**

Physical pain was one of the most common experiences reported by participants, significantly affecting the enjoyment of life after discharge.

*- " My right shoulder and pelvis hurt so much that I couldn’t lie on my right side for the first month. The pain was so intense that I felt hopeless about life” (P17, 31-year-old woman).*

The coexistence of pain and the inability to perform personal tasks led to feelings of helplessness. Exaggerated interpretations of the situation, unrealistic fears about pain severity, and perceived dependence on others were reported in participants’ experiences.

*- “After returning home, I was bedridden for two full weeks and couldn’t perform my own care. I gradually began to believe that I was a helpless person who would be damaged and destroyed if others were not around for a moment.” (P.4, 42-year-old man)*

*- “After discharge, my wife fed me. I could not even bathe without assistance. From the contemptuous looks of others, I realized that I always had to rely on others to perform these personal activities.” (P4, 42-year-old man)*

**4.3. Feeling guilty about not fulfilling roles**

Feelings of helplessness and physical weakness led most participants to perceive themselves as unable to fulfill their usual roles, viewing their responsibilities as incomplete or neglected. As a result, their tasks were transferred to other family members, which in turn generated a sense of guilt due to the perceived negative impact on family life.

*- “I didn’t do anything for my children. When I think about it, I feel guilty, like they don’t have a mother. Family duties fell on my husband.” (P.9, 45-year-old woman)*

*- “I couldn’t do anything for my children. When I think about it, I feel bad, as if they didn’t have a mother” (P15, 63-year-old woman).*

**4.4. Loss of speech and altered appearance as barriers to social interaction**

Participants described two conditions as essential prerequisites for social interaction: the ability to communicate verbally in a clear and fluent manner, and possessing an acceptable or aesthetically pleasing appearance. Alterations in these two areas negatively affected their willingness to interact with others or appear in public.

*- “In the early days after discharge, my face had changed; I had no hair, and my throat was open because of the tracheostomy. In that condition, I didn’t want anyone to see me or hear my voice, so I told my mother that no one except her and my husband was allowed into my room.” (P.7, a 32-year-old woman)*

*- “My voice had become altered and muffled due to throat swelling and the thoracostomy opening; therefore, I did not like to speak much in family gatherings.” (P13, 25-year-old man)*

## Discussion

As the findings of the present study revealed, the post- discharge life experience of trauma patients is a multifaceted phenomenon comprising both pleasant and distressing elements. The category “Freedom from the captivity of alienation and despair” reflects the unpleasant emotional burden associated with ICU hospitalization. This ambiguous and unsettling experience stemmed largely from patients’ lack of awareness regarding their condition during the period of unconsciousness and their confusion after regaining consciousness. These findings highlight the critical need for structured nursing interventions aimed at reassurance and continuous communication with comatose ICU patients, even when they are not consciously responsive. These results are in line with a study, which emphasized that ICU nurses must continuously enhance their communication competencies when caring for critically ill and comatose patients. According to their findings, effective communication during ICU stay is not only essential for reducing patients’ psychological distress but is also directly associated with the quality of post-discharge care and recovery outcomes.^17^ Based on participants’ experiences, nurses should engage in clear and continuous communication with ICU patients about the reason for admission, anticipated length of stay, upcoming nursing procedures—even during unconsciousness—and likely discharge timing. Providing such information can reduce confusion upon regaining consciousness and alleviate feelings of alienation and disorientation. Nurses should reconsider the common assumption that unconscious patients cannot understand what is being said, as ongoing verbal reassurance and explanation may enhance psychological comfort and a sense of safety.^18^ In a review study, it was reported that approximately 25–40% of patients with impaired levels of consciousness are able to hear and comprehend conversations in their surroundings.^19^ This evidence reinforces the necessity for nurses to communicate verbally with patients even during periods of unconsciousness.

The present study also revealed that although the post-discharge life experience was described as pleasant, it was concurrently accompanied by persistent concerns about potential ICU readmission. This finding underscores the importance of effective communication and post-discharge follow-up to address ongoing challenges. Previous research has shown that approximately 50% of ICU survivors develop new physical, psychological, and/or cognitive problems after discharge.^20^ These new post-discharge problems, combined with fear of the unfamiliar ICU environment, make the prospect of readmission particularly daunting for patients. Providing comprehensive support during ICU hospitalization, along with structured physical and psychological rehabilitation after discharge, can help patients perceive the potential experience of readmission as more manageable. A previous study indicated that most factors contributing to ICU readmission are related to the actions of nurses and physicians, highlighting the need to improve the quality of care, while patients themselves have a limited role in influencing these outcomes.^21^

In our study, the category “Integration of Real and Imagined Disability and Helplessness” was identified. This category emphasizes that attention should be given not only to patients’ actual functional limitations after discharge but also to their unrealistic perceptions of their own abilities. In a study reported that ICU patients experience real physical and psychological problems after discharge, which affect their self-perception, family interactions, and ability to resume daily life.^22^ Another study found that a significant proportion of ICU survivors suffer from distorted memories and exaggerated interpretations of their experiences.^23^ Similarly, a previous study noted that ICU hospitalization, accompanied by physical and mental suffering, constitutes an unpleasant experience.^12^ The findings of our study are consistent with these reports, highlighting the importance of attending to both physical and psychological disorders even after discharge. However, unlike previous studies, our results indicate that some post-discharge physical and psychological responses may arise from patients’ exaggerated perceptions of their situation. We labeled this aspect as “imagined disability and helplessness.” These findings suggest that future home care planning should address not only real impairments but also patients’ subjective and potentially exaggerated perceptions of their limitations following recovery from coma.

**Limitations**

Despite our efforts to provide a detailed description of the post-discharge life experience among trauma patients recovering from coma, several limitations should be noted. First, we focused solely on patients’ self-reported experiences; incorporating additional data sources, such as patient diaries or nursing notes, could have provided a more comprehensive understanding of the phenomenon. Second, due to the qualitative nature of the study, data collection relied exclusively on interviews. While interviews offered rich insights, direct observation might have further enhanced the depth and validity of the findings in future research.

## Conclusion

The initial perception of post-discharge life among trauma patients who recovered from coma is profoundly shaped by the unpleasant experiences endured during ICU hospitalization. The sufferings experienced at home reflect a combination of real complications and imagined concerns arising from the severity of trauma, often accompanied by a false sense of disability and helplessness. These findings underscore the necessity of softening the ICU environment, implementing comprehensive discharge preparation, and providing effective post-discharge follow-up that addresses not only actual physical and psychological problems but also perceived or imagined sufferings.

**Acknowledgements**

We sincerely thank all participants for sharing their valuable experiences. We also acknowledge the support of Golestan Hospital, Ahvaz Jundishapur University of Medical Sciences, in facilitating the conduct of this study.

**Authors' contributions**

Conceptualization and study design: Mr. Imani, Dr. Molavynejad, Dr. Khademi, and Dr. Adineh. Data Gathering: Mr. Imani. Data Interpretation: Mr. Imani, Dr. Molavynejad, Dr. Khademi, and Dr. Adineh, and Dr. Savaie. Manuscript Draft Preparation: Mr. Imani, Dr. Molavynejad, Dr. Khademi, and Dr. Adineh.
